# Comparative Phytoprofiling of *Achillea millefolium* Morphotypes: Assessing Antioxidant Activity, Phenolic and Triterpenic Compounds Variation across Different Plant Parts

**DOI:** 10.3390/plants13071043

**Published:** 2024-04-08

**Authors:** Lina Raudone, Gabriele Vilkickyte, Mindaugas Marksa, Jolita Radusiene

**Affiliations:** 1 Department of Pharmacognosy, Lithuanian University of Health Sciences, Sukileliu Avenue 13, 50162 Kaunas, Lithuania; 2 Laboratory of Biopharmaceutical Research, Institute of Pharmaceutical Technologies, Lithuanian University of Health Sciences, Sukileliu Avenue 13, 50162 Kaunas, Lithuania; gabriele.vilkickyte@lsmu.lt; 3 Department of Analytical and Toxicological Chemistry, Lithuanian University of Health Sciences, Sukileliu Avenue 13, 50162 Kaunas, Lithuania; mindaugas.marksa@lsmu.lt; 4 Laboratory of Economic Botany, Nature Research Centre, Akademijos Street 2, 08412 Vilnius, Lithuania; jolita.radusiene@gamtc.lt

**Keywords:** *Achillea millefolium*, morphotypes, caffeoylquinic acid, betulinic acid, radical scavenging activity, reducing activity

## Abstract

*Achillea millefolium* L., commonly known as yarrow, is a versatile and widely distributed plant species with a rich history of ethnopharmacological significance. This study aimed to evaluate the comparative differences of *A. millefolium* inflorescence morphotypes. The phytochemical profile of white and pink inflorescence morphotypes was characterised by a complex of thirty-four phenolic and triterpene compounds. The species has distinct morphotypes of white and pink inflorescence. Phenolic and triterpenic profiles were determined, and individual compounds were quantified in inflorescence, leaf, and stem samples of two morphotypes tested. The antioxidant activity of plant extracts was evaluated by free radical scavenging (ABTS) and ferric-reducing antioxidant power (FRAP) assays. Caffeoylquinic acids predominated in all parts of the plant tested. Chlorogenic acid and 3,5-dicaffeoylquinic acid were the principal compounds in the phenolic profile. Betulin, betulinic acid, and α-amyrin were the prevailing triterpenic components in the triterpenic profiles of *Achillea millefolium* morphotypes. The predominant flavonoids in inflorescences were flavones, while in leaves, flavonols were the organ-specific compounds. The quantitative differences were observed between plant parts of morphotypes. Leaves consistently displayed the highest amounts of identified compounds and have been testified as the main source of antioxidant activity. Overall, white inflorescences accumulated a higher total amount of compounds compared to pink ones. The observed differences between morphotypes derived from the same population reflect the differences in specialised metabolites and their chemotypes. This study addresses gaps in knowledge, particularly in phenolic and triterpenic profiling of coloured inflorescence morphotypes, enhancing our understanding of chemotypes and morphotypes within the species.

## 1. Introduction

As the prevalence of chronic diseases continuously increases, there is a rising interest in the research of natural compounds and their sources, providing substantial challenges for the pharmaceutical industry. Additionally, there is a growing focus on using medicinal plants with profound antioxidant activity to enhance the value, safety, and application of products in many other fields, such as nutraceuticals, foods, and cosmetics [1,2,3,4]. Moreover, there is still a significant gap in understanding the pharmacological potential of plant extracts from complex botanical matrices and coupling the morphological and phytochemical markers to bioactivity markers. Using herbs as medicine is essential to human history and cultural heritage. 

One of the oldest known plants used by humans for thousands of years and considered ethnopharmacologically relevant is *Achillea millefolium* L. [5,6]. *Achillea millefolium s.l.*, a member of the Asteraceae family, includes a group of closely related taxa commonly known as yarrows, widespread throughout the temperature and boreal zones of the Northern Hemisphere [7,8]. These are hardy perennial herbs, about 30–60 cm high, with feathery leaves and clusters of small, white, pink or reddish flowers with a strong aroma and bitter taste [6,9,10]. The taxonomy of *A. millefolium* is highly complicated due to its infraspecific variability, hybridisation, genetic polymorphism and ecological plasticity, and there is no single accepted classification of the *A. millefolium* complex [8]. 

Studies on the chemical constituents of *A. millefolium* showed the presence of a wide range of bioactive compounds, namely mono- and sesquiterpenoids, lactones, phenolic compounds, amino acids, fatty acids, organic acids, sugars, saponins, and coumarins. Among them, flavonoids (derivatives of apigenin and luteolin), caffeoylquinic acids, and essential oils monoterpenes were suggested as the most representative metabolites associated with health benefits [5,11,12,13]. Due to its safety and multifunctionality, *A. millefolium* has many applications in medicine, veterinary science, and cosmetics. It has been used in the treatment and management of pains, bleeding, dermatological, inflammatory, hepatobiliary, cardiovascular, respiratory, and gastrointestinal disorders, as well as diabetes and neurodegenerative diseases regarding the antioxidant properties and related anti-ageing and rejuvenation mechanisms of active ingredients [9,14,15,16,17]. 

Regarding medicinal plants’ safety and efficacy assurance, phytochemical standardisation and reproducibility are important. The genetic factors, climatic conditions, growing environment, origin, and phenological stage were considered to affect the chemical composition of plants [18,19]. Changes in abiotic conditions can lead to differences in the pharmacological and therapeutic properties of wild plant materials and determine the value and quality of the derived products. Observations of phytochemical content in plant raw materials under the influence of relevant factors can help to understand and control the quality of plant material by selecting the most promising plant wild clones and favourable growth conditions to determine the highest levels of targeted bioactive compounds [20,21,22]. 

Previous studies have mainly focused on the characterisation of essential oils and flavonoid components of the typical white morphotype of *A. millefolium* in relation to external and internal cues [13,23,24,25,26]. However, to date, no study has reported a detailed investigation of the triterpenoid profile in *A. millefolium* parts of the plant. Since triterpenic compounds have many potential applications and a broad spectrum of pharmacological activities [27,28], this study would substantially complement the comprehensive phytochemical characterisation of bioactive compounds in *A. millefolium* and would indicate a possible contribution of triterpenoids to the potent antioxidant potential. As far as we are aware, this is the first study to report the composition of triterpenoids and phenolic compounds as well as antioxidant activity in inflorescences, stems, and leaves of two different *A. millefolium* morphotypes with white (W) and pink (P) inflorescences. The research couples the assessment of triterpenoids, a relatively unexplored area, with phenolic compounds and antioxidant activity. This study aimed to evaluate the phytochemical composition of *A. millefolium* white and pink inflorescence morphotypes and to provide insights into the accumulation trends of phenolic and triterpenic compounds across morphotypes and parts of the plant in relation to the potential of antioxidant activity. The results of the comparative study would have an indicative function, contribute to the assessment of genetic and geographical diversity data, and support the integrated use of different parts of *A. millefolium*.

## 2. Results

### 2.1. Phenolic and Triterpenic Profiles of Two Morphotypes: Inflorescences, Leaves and Stems 

The complex of thirty-four phenolic and triterpenic compounds was identified in the inflorescences, leaf, and stem samples of two *A. millefolium* morphotypes (Table 1). The phenolic profile was principally composed of caffeoylquinic acids and flavones, namely luteolin and apigenin glycosides. The flavonols were minor compounds, and their variability strongly depended on the tested plant materials. The triterpenic profile was comprised of triterpenic acids (oleanolic acid, ursolic acid, maslinic acid, corosolic acid, betulinic acid), neutral triterpenoids (betulin, uvaol, α-amyrin, β-amyrin), phytosterols (β-sitosterol), and triterpene esters (betulinic acid methyl ester). Betulin, betulinic acid, and α-amyrin were the prevailing triterpenic compounds in all samples tested. 

Ten quantitatively prevailing (>1000 µg/g, DW) compounds belonging to the groups of caffeoylquinic acid, flavone glycosides, and neutral triterpenoids were determined in the samples of *A. millefolium* inflorescences. Chlorogenic acid and 3,5-*O*-dicaffeoylquinic acid were the predominant compounds with the highest amounts (*p* < 0.05) found in all the tested inflorescence samples of white (7969.08 ± 226.96 µg/g and 9014.24 ± 732.64 µg/g, respectively) and pink (7848.88 ± 346.64 µg/g and 8502.93 ± 762.67 µg/g, respectively) morphotypes. 3,5-*O*-Dicaffeoylquinic acid prevailed over chlorogenic acid in all inflorescence samples tested. The amounts of other major compounds ranged from 1000 to 3600 µg/g, DW. However, the quantitative differences between the predominant compounds in W and P morphotype samples were not detected, with the exception of significantly higher levels of betulin and 4-*O*-caffeoylquinic acid in white inflorescences. The amounts of other prevailing compounds were higher in W inflorescences, although no significant differences were found. The amounts of other identified compounds were in a range of 8–760 µg/g. Significant differences between the amounts of compounds in W and P inflorescences were determined only for triterpenic compounds, namely oleanolic acid, maslinic acid, corosolic acid, betulinic acid, β-amyrin, and α-amyrin, with greater (*p* < 0.05) amounts in P and W inflorescences, respectively. Overall, the total amount of identified compounds in W inflorescences was higher compared to P inflorescences, 39,680.41 µg/g and 37,193.72 µg/g, respectively.

Leaves of both *A. millefolium* morphotypes contained the greatest (*p* < 0.05) amounts of identified compounds compared to inflorescences and stems. Thirty-three compounds’ phytochemical profiles were characterised. The qualitative profile corresponded with that of inflorescences, except that leaf samples additionally contained ursolic acid, nicotiflorin, isoquercitrin and cynarin, which were absent in inflorescences. The total amount of identified compounds did not show significant differences between the leaves of W and P morphotypes and accounted for 71,657.67 and 70,748.81 µg/g, respectively. The quantitatively prevailing compounds were phenolic compounds with the highest amounts of chlorogenic acid—28,123.17 ± 1013.01 µg/g and 28,934.28 ± 1864.98 µg/g in W and P morphotypes, respectively (Table 1). The prevailing triterpenic compounds in the leaves were betulin (956.93 ± 110.63 µg/g and 992.32 ± 153.83 µg/g in W and P, respectively) and betulinic acid (440.54 ± 78.94 µg/g and 519.01 ± 51.77 µg/g in W and P, respectively). The amounts of individual compounds varied among leaf samples of morphotypes, showing compound-specific predominance in the respective W or P morphotype of *A. millefolium*. The leaves of both W and P morphotypes accumulated the highest total amount of determined phenolics compared to inflorescences and stems. Furthermore, the total amount of triterpenic compounds was consistent in leaf and inflorescence samples, with the highest total amount in W inflorescences (*p* < 0.05). Moreover, the key differences in the phenolic profiles in inflorescence and leaf samples were between flavone and flavonol composition; although no significant differences were found between morphotypes for the total amount of compounds, flavones predominated in inflorescences and flavonols in leaves. Consequently, the leaves and inflorescences have their plant organ-specific profiles with a characteristic composition of triterpenic and phenolic compounds. 

The total amount of identified compounds in W and P stems was six-fold lower than in inflorescence and leaf samples. Stems accumulated the lowest amounts (*p* < 0.05) of all identified compounds. Chlorogenic acid, 3,5-dicaffeoylquinic acid, 1,5-caffeoylquinic acid, and rutin were predominant phenolics found in the stems (Table 1). Betulin pre-vailed in the triterpenic profile, accounting for up to 83 and 84% of total triterpenic compounds in stem samples of P and W morphotypes, respectively. The quantitative profile of flavones and flavonols corresponded to the profile of leaves with a significant predominance of flavonols (*p* < 0.05). The stems were distinguished by the absence of some minor compounds, such as oleanolic acid, ursolic acid, betulinic acid, uvaol, nicotiflorin, isovitexin, and apigenin, which were found in other parts of the plant (Table 1).

The phytochemical composition of phenolic and triterpenic compounds of *A. millefolium* was not appreciably affected by habitat. The analysis did not reveal statistical differences in the amounts of chemical compounds in inflorescences, leaves, and stems between different populations. The pink and white morphotypes exhibited different phytochemical profiles, but these differences were morphotype-dependent and were not influenced by the growing site of populations. Overall, the study suggests that the intricate chemical makeup of *A. millfolium* is mainly determined by internal factors, such as morphotype, rather than external environmental conditions. 

### 2.2. Principal Component Analysis

Principal component analysis (PCA) was applied to determine the phytochemical relationships between morphotypes in individual plant organs. Individual phenolic and triterpenic compounds and their phytochemical groups were used as variables to create two-dimensional PCA matrix models to visualise the available plant organ-specific patterns of two *A. millefolium* morphotypes. 

**Figure 1:**
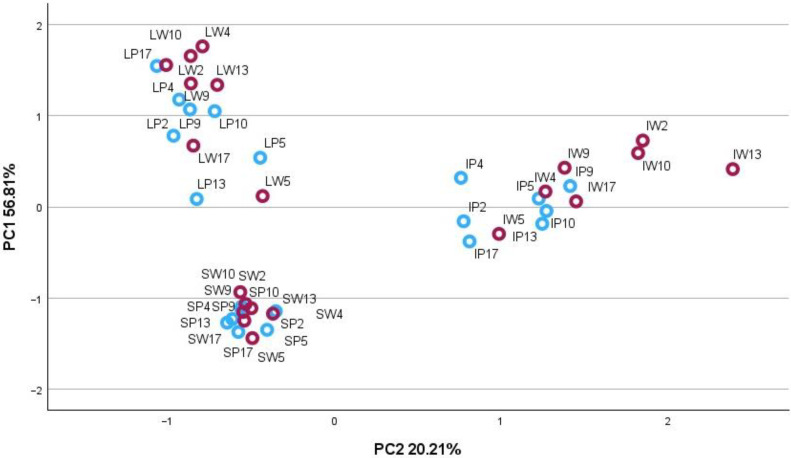
PCA-1 score plot model presenting the amounts of predominant phenolic and triterpenic compounds in plant organs (I−inflorescences; L−leaves; S−stems) of P (blue circles) and W (red circles) of *A. millefolium* morphotypes.

The correlation matrix for the variables and principal components of the PCA-1 model accounted for 77.02% of the total variance of the data set of inflorescence, leaf, and stem samples (Figure 1). The PC1 explained for 56.81% of the total variance and was highly correlated with β-sitosterol (0.915), α-amyrin (0.637), luteolin-7-glucuronide (0.882), chlorogenic acid (0.858), 3,4-dicaffeoylquinic acid (0.964), 3,5-dicaffeoylquinic acid (0.937), 1,5-caffeoylquinic acid (0.750), 4,5-dicaffeoylquinic acid (0.854), and luteolin-7-rutinoside (0.801). The PC2 explained 20.21% of the total variance and was positively correlated with betulin (0.875), β-amyrin (0.721), α-amyrin (0.560), luteolin (0.696), luteolin-7-glucoside (0.848), apigenin (0.958), apigenin-7-glucoside (0.956) and negatively with rutin (—0.687). The score plot showed the arrangement of plant organ samples of W and P *A. millefolium* morphotypes (Figure 1). All samples fell into three distinct plant organ-specific groups of overlapping morphotypes. On the other hand, inflorescence samples tended to group into clusters, coupling the distinct morphotypes. Therefore, the inflorescences were subjected to the PCA-2 model.

**Figure 2:**
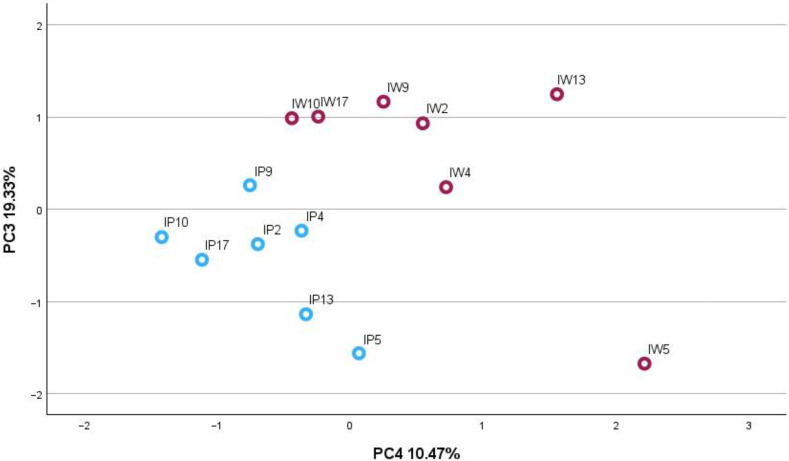
PCA-2 score plot model presenting the amounts of predominant phytochemical compounds in inflorescences (I−inflorescences) of P (blues circles) and W (red circles) *A. millefolium* morphotypes.

The PCA-2 model correlation matrix was constructed for inflorescence samples using triterpenic compounds (Figure 2) and covered 85.56% of the total variance, resulting in four components. The PC1 explained 31.51% of the total variance in the data set and was highly correlated with corosolic acid (0.930), betulin (0.806), and uvaol (0.854). The PC2 accounted for 24.25% of the total variance and was correlated with oleanolic acid (0.821), ursolic acid (0.924), and maslinic acid (0.823). The PC3 described 19.33% of the total variance and was correlated with betulinic acid methyl ester (0.870), β-amyrin (0.807), and β-sitosterol (0.701). The PC4 presented 10.47% of the total variance and was correlated with betulinic acid (0.970) and α-amyrin (0.922). The plotting of PC3 and PC4 presented the best separations between the pink and white morphotype samples. The inflorescences of P *A. millefolium* were positioned on the positive side of the scatterplot of PC3 and PC4, indicating the highest levels of α, β-amyrins, β-sitosterol, betulin, and betulinic acid methyl ester compared to W samples. 

**Figure 3:**
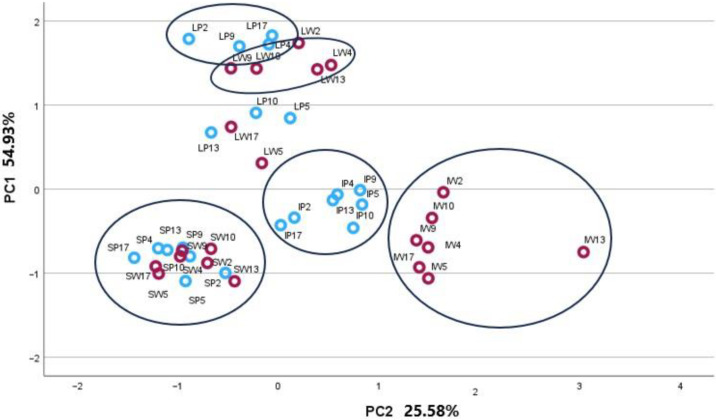
PCA-3 score plot model presenting phytochemical compound groups in plant organs (I−inflorescences; L−leaves; S−stems) of P (blue circles) and W (red circles) *Achillea millefolium* morphotypes.

The PCA-3 model was constructed to identify the variance in concentrations of specialised metabolite groups in plant organs and morphotypes. Groups of chemical compounds of similar origin were as follows: sum of titerpenic compounds, sum of triterpenic acids, sum of neutral triterpenes, sum of phytosterols, sum of triterpene esters, sum of phenolic compounds, sum of flavonoids, sum of flavones, sum of flavonols, sum of flavanones, and sum of phenolic acids. The matrix accounted for 80.50% of the total variance of the data set. The PC1 explained 54.93% of the total variance and was correlated with the sum of triterpenic compounds (0.824), the sum of phenolic compounds (0.918), the sum of flavonoids (0.601), the sum of phenolic acids (0.942), the sum of flavonols (0.836), and the sum of flavanones (0.946). The PC2 presented 25.58% of the total variance and was correlated with the sum of triterpenic acids (0.896), the sum of neutral triterpenes (0.839), the sum of phytosterols (0.676), the sum of flavonoids (0.718), and the sum of flavones (0.856). The arrangement of inflorescence and leaf samples in PC1 and PC2 plot space revealed their clustering according to P and W morphotypes, although the stem morphotypes remained in an overlapping position (Figure 3). 

Overall, the distant position towards the positive PC2 axis demonstrated the unique peculiarities of the P morphotype inflorescences. Pink inflorescences contained more triterpenic acids, neutral triterpenes, and phytosterols and were marked by high levels of flavones. Several leaf samples tended to cluster by morphotypes, as W samples showed higher amounts of total triterpenic compounds and flavonoids than P ones. Stem samples were positioned at the negative sides of both components and indicated the lowest amounts of all compounds.

### 2.3. Assesment of Radical Scavenging and Reducing Activity of Achillea millefolium Extracts

**Figure 4:**
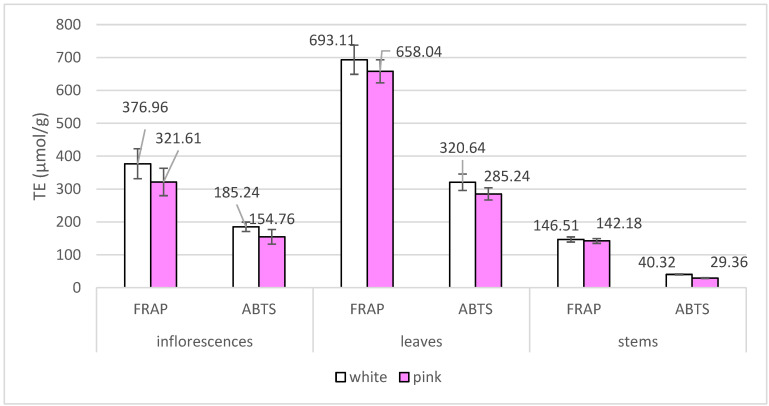
Antioxidant activity mean Trolox equivalent (TE) values (µmol/g, DW) of inflorescences, leaves and stems of white and pink *A. millefolium* morphotypes.

The antioxidant activity of *A. millefolium* morphotypes was evaluated using two action assays: Ferric Reducing Antioxidant Power (FRAP) and ABTS radical cation decolourization assay (ABTS). The results are presented in Figure 4.

Antioxidant activity values varied significantly between different plant organs tested (*p* < 0.05). The highest values of radical scavenging and reducing activity were determined for leaf samples of both morphotypes. The mean FRAP values for the pink morphotype were 321.61, 658.04, and 142.18 µmol TE/g for inflorescences, leaves, and stems, respectively. The corresponding ABTS values were 154.76, 285.24, and 29.36 µmol TE/g. The results indicate that the samples of white *A. millefolium* morphotype exhibit higher antioxidant activity in all three parts of the plant studied as measured by both FRAP and ABTS assays. The antioxidant activity of inflorescences was intermediate, while stems showed the lowest activity, within the range of their variation. These findings suggest that the leaves are the principal source of antioxidant active compounds. The detailed phytochemical analysis of bioactive compounds could contribute to understanding the underlying factors determining these differences. The correlations were established between the antioxidant values and identified compounds. Compounds, namely oleanolic, ursolic, maslinic, corosolic acid, uvaol, β-sitosterol, nicotiflorin, isovitexin, hesperidin, luteolin-7-glucuronide, neochlorogenic acid, chlorogenic acid, cryptochlorogenic acid, 3,4-dicaffeoylquinic acid, 3,5-dicaffeoylquinic acid, 1,5-caffeoylquinic acid, 4,5-dicaffeoylquinic acid, caffeic acid, quercitrin, rutin, isoquercitrin, luteolin-7-rutinoside, santin, and cynarin showed a positive correlation (*p* < 0.0001) varying in the range of 0.527–0.824 and 0.577–0.923 for the radical scavenging and reducing activities, respectively. The correlation between the total number of determined phenolic compounds was 0.815 and 0.946 (*p* < 0.0001) for ABTS and FRAP assays, respectively. Furthermore, the correlation between the total amount of triterpenic compounds detected and the ABTS and FRAP assay results was found to be 0.769 and 0.841, respectively.

## 3. Discussion

The exploration of *A. millefolium* is still relevant because it represents a plant species of considerable ethnopharmacological and classical medicinal importance [29]. Our specific focus on *Achillea* species in this context is driven by the potential therapeutic benefits and ecological significance associated with the diverse compounds present in this plant, making it a valuable subject for comprehensive investigation. Although literature data covers various aspects of the phytochemical profiles, there are still undescribed aspects that would add to the scientific knowledge of *A. millefolium* phytochemical maps [5]. The plant’s ability to grow in different climates and soil types has contributed to its successful establishment in various geographic regions around the world [3]. Our previous study assessed the phytochemical diversity of *A. millefolium* along geographical gradients. The content of phenolic compounds in yarrow raw material from northern latitudes was found to be more than twice as high as that of southern latitudes, potentially reflecting greater plant adaptation to the respective conditions. 

The present study encompasses the colour of flowers used as a differential intraspecious character to discriminate morphotypes as taxa of *A. millefolium*. The morphological diversity of *A. millefolium* covers various morphological characters, the most prominent of which is the different colours of flowers. In this context, a previous morphological study of wild *A. millefolium* showed that among 3345 evaluated plants from 147 cenopopulations, plants with white inflorescences accounted for 78.8% and the rest pink ones [30]. Furthermore, a correlation was found between the inflorescence colour and other morphological characters, which indicated that the colour of inflorescences can be used as a marker to identify intraspecific taxa [30]. In this way, when assessing the diversity potential of yarrow, it was important to find out the relationship between flower colour and the phytochemical composition of the plant material. In this case, chemophenetics is useful for coupling chemotypic and morphotypic variation [31]. Our investigations reveal some variations in the underlying phytochemical profiles of the inflorescence colour-specific morphotypes. Available literature data suggested that the white-flowered *A. millefolium* morphotype exhibited a different accumulation of certain compounds, such as flavonoids and essential oils, compared to its pink-flowered morphotype [32]. Garzoli et al. found that the aerial parts of the pink morphotype *A. millefolium* accumulated higher amounts of monoterpenes than the white morphotype [33]. According to the authors, these differences in phytochemical composition between morphotypes suggested a potential adaptation to different ecological conditions or reproductive strategies. However, this assumption contradicts our findings, as white and pink plant samples were collected from the same mixed stands, eliminating the influence of habitat conditions on morphotype diversity. Thus, the observed phytochemical diversity of *A. millefolium* morphotypes potentially reflected genetic diversity. The colour of the inflorescences can be considered an indicator of a certain chemotype of *A. milllefolium* plant material. 

Our findings revealed that the caffeoylquinic acid complex predominantly shaped the phenolic profile in both pink and white morphotypes of *A. millefolium*. Mono- and dicaffeoylquinic acids are fast-acting antioxidants and contribute to the total antioxidant activity of plant extracts [5,34]. Moreover, they are also considered the key compounds of hepatoprotective and choleretic activity [35]. *Achillea* spp. inflorescences were characterised by flavones as organ-specific compounds, while leaves were characterised by flavonols, especially rutin, which is in agreement with our previous studies [23,36]. *Achillea* spp. flavonoids have been linked with antispasmodic, anti-ulcer, antiproliferative, estrogenic, antioxidant, and anti-inflammatory properties [5]. Antioxidant and anti-inflammatory properties are closely related and play a significant role in the pathogenesis of chronic degenerative diseases [9]. Determining the antioxidant activity of plant extracts is crucial to elucidate their therapeutic and nutritional benefits, as antioxidants play a key role in mitigating oxidative stress. There is no universal method that can accurately assess antioxidant capacity and obtain an unambiguous result. Applying assays with diverse modes of action can provide a deeper understanding of the action of chemical compounds and their potential to counteract oxidative stress through varied mechanisms [37]. Antioxidants can potentially influence the activity of transcription factors related to the immune response, triggering the reduction of pro-inflammatory cytokines [38]. Antioxidants in target sites are capable of free radical quenching or oxidation chain termination, thus participating in cancer chemoprevention [39]. However, significant variations in the concentrations of phenolic compounds and total antioxidant activity were observed between the morphotypes and parts of the plant. These variations highlight the dynamic and adaptive nature of the plant in response to its local environment. Plants alter the phenolic composition and antioxidant capacities of their tissues as an adaptive response to regulate environmental stresses [40]. 

The analysis of triterpenic profiles uncovered the presence of several biologically active compounds, including oleanolic acid, maslinic acid, corosolic acid, betulinic acid, betulin, uvaol, betulinic acid methyl ester, β-amyrin, β-sitosterol, and α-amyrin. The concentrations of these triterpenic compounds exhibited variability in the tested samples, with potential implications for the medicinal properties of *A. millefolium* raw material. The available data on compounds of triterpenic origin in the *Achillea* spp. are scarce. Fifteen compounds of triterpenic origin, including α-amyrin, β-amyrin, and β-sitosterol, were previously identified in *A. alexandri-regis* [41], while magnificol, β-sitosterol, α-amyrine, and lupeol were detected in *A. magnifica*. In the methanolic extracts of flowering parts of *A. ligustica*, the triterpene moretenol was detected as the principal compound, followed by stigmast-6-en-3β-ol, veridiflorol, and β-amyrin [42]. Betulin was identified as the essential oil of endemic *A. eriophora* from Iran [43]. In addition, four sterols, β-sitosterol, cholesterol, campesterol, and stigmasterol and α and β-amyrins, have been reported in *A. millefolium* [44]. A fraction of *A. millefolium* rich in stigmasterol and β-sitosterol has been shown to have skin-calming and anti-inflammatory effects [39]. In addition, triterpene components have significant anti-inflammatory activity and may also contribute to specific pharmacological activity with implications for inflammation and cancerogenesis [45,46]. However, most of the studies were performed on the whole areal part of *Achillea* spp., without dissecting it into individual plant organs. Studies of individual plant organs allow us to reveal a more targeted use of plant material. In this regard, the leaves of the morphotypes revealed a qualitatively different profile with a characteristic composition of triterpene and phenolic compounds, indicating their additional value. Hence, in the pharmaceutical raw material of *A. millefolium*, which is defined and used both in the European pharmacopoeia and in folk medicine as a herb (*Millefolii herba*) or flowers (*Millefolii flos*) [47,48], leaves with additional value, should be given more attention.

In the current study, phytochemical differences between individual plant organs and morphotypes of *A. millefolium* were determined using PCA, which highlights the morphotype-dependent variations in phytochemical profiles. This separation is particularly pronounced regarding triterpenic acids, neutral triterpenes, and phytosterols, emphasising the unique chemical makeup of morphotype inflorescences. This work has expanded our understanding of bioactive compounds and their chemical diversity in *A. millefolium* inflorescence morphotypes, which may contribute to the additional application of this medicinal plant. Differences in specialised metabolites and their chemotypes likely reflect their genetic differences, as both morphotypes originated from the same population. The morphotype can serve as a marker for the selection of a certain chemotype.

To the best of our knowledge, no data have been found regarding phenolic and triterpenic profiling in coloured inflorescence morphotypes of *A. millefolium*. Furthermore, the databases do not contain information on the pigment composition of coloured inflorescences of *A. millefolium* morphotypes. Given these gaps in knowledge, future research could focus on investigating the triterpenic profiles and anthocyanin content of *Achillea* spp. inflorescences, particularly focusing on flower colour variations.

## 4. Materials and Methods

### 4.1. Chemicals and Reagents

The following analytical or chromatographic grade solvents were used in the study: 99.9% acetonitrile, 99.9% methanol, 99.8% anhydrous acetic acid, and 37% hydrochloric acid from Sigma-Aldrich (Steinheim, Germany); 99.8% trifluoroacetic acid from Merck (Darmstadt, Germany); and purified water was prepared using Milli–Q (Millipore, Bedford, MA, USA) water purification system. The following high purity substances were used: rutin, santin, quercetin, quercetin-3-*O*-glucoside, nicotiflorin (kaempferol-3-*O*-rutinoside), luteolin, luteolin-7-*O*-glucoside, luteolin-7-*O*-rutinoside, luteolin-7-*O*-glucuronide, apigenin, apigenin-7-*O*-glucoside, 3-*O*-caffeoylquinic acid (chlorogenic acid), 5-*O*-caffeoylquinic acid (neochlorogenic acid), 4-*O*-caffeoylquinic acid, 1,3-*O*-dicaffeoylquinic acid (cynarin), 3,4-*O*-dicaffeoylquinic acid, 3,5-*O*-dicaffeoylquinic acid, 1,5-*O*-dicaffeoylquinic acid, 4,5-*O*-dicaffeoylquinic acid, α-amyrin, β-amyrin, β-sitosterol, maslinic acid, and oleanolic acid from Sigma-Aldrich; uvaol, betulin, betulinic acid, corosolic acid, and betulinic acid methyl ester from Extrasynthese (Genay, France); ursolic acid from Carl Roth (Karlsruhe, Germany). The following reagents for antioxidant activity determination were used: 6-hydroxy-2,5,7,8-tetramethylchroman-2-carboxylic acid (Trolox), 2,2′-azino-bis(3-ethylbenzothiazoline-6-sulfonic acid) diammonium salt (ABTS), 2,4,6-tri-(2-pyridyl)-S-triazine (TPTZ), ferric chloride (FeCl_3_), and sodium acetate from Sigma-Aldrich (Steinheim, Germany); and potassium persulfate from Alfa Aesar (Karlsruhe, Germany). 

### 4.2. Plant Material

**Figure 5:**
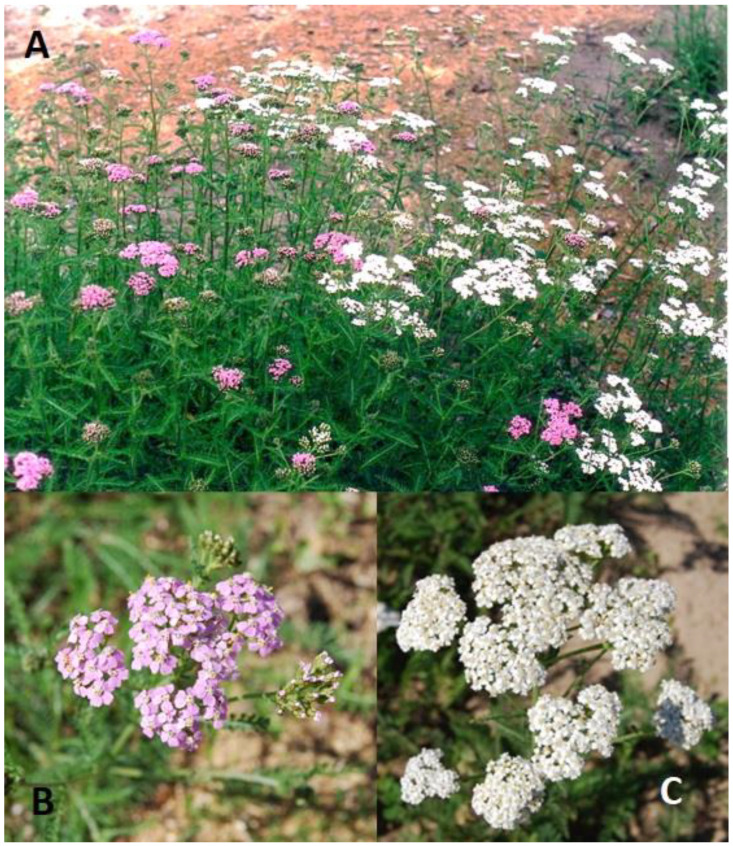
Two wild morphotypes of *Achillea millefolium* ((**A**)—mixed stands; (**B**)—pink morphotype; (**C**)—white morphotype). Photo by authors.

Aerial parts of the plant of two *A. millefolium* morphotypes with white (W) and pink (P) inflorescences (Figure 5) were sampled from seven mixed morphotype stand populations (Table 2) during full bloom in June and August 2022. Plant material consisting of 30 single shoots per sample was dissected into inflorescences, leaves, and stems and then dried in a drying cabinet type SSO-80 (Isoterma, Wroclaw, Poland) at 25 °C temperature and 10% relative humidity for 24 h. The botanical identification of species was performed on morphological characters according to descriptors [7,47]. 

The air-dried plant material samples were ground to homogenous powder using a Retsch 200 mill (Haan, Germany); the material was crushed to particles passing through a 355 μm sieve and kept in the dark in sealed containers until extraction. 

### 4.3. Preparation of Plant Extracts

Extraction of phenolic compounds was performed as follows: 0.1 g of fine powder of dried stems, leaves, or inflorescences were extracted with 10 mL of 70% *v*/*v* methanol in water for 30 min at 40 °C in an ultrasonic bath Elmasonic P (Singen, Germany). Extraction of triterpenoids was performed by extracting 1 g of powdered raw material with 10 mL of pure methanol using ultrasound-assisted extraction for 25 min. All obtained extracts were filtered through 0.22 µm pore size membrane filters (Carl Roth GmbH, Karlsruhe, Germany) and stored at 4 °C until analyses.

### 4.4. HPLC-PDA Conditions

The HPLC-PDA (Waters e2695 Alliance system, Milford, MA, USA) system was used to analyse phenolic and triterpenic compounds. The identification was performed by comparing retention times and spectra of samples to those of commercially available standard substances. The concentration of each compound was calculated based on the external standard method using linear regression models. Quantitative phytochemical profiles were expressed as the mean and standard deviation (SD) from three replicates.

For phenolic compounds analysis, ACE Super C18 (250 mm × 4.6 mm, 3 µm) reversed-phase column (ACT, Aberdeen, UK), maintained at 35 °C, was used. The gradient consisting of eluent A (0.1% trifluoroacetic acid in water) and eluent B (100% acetonitrile) was formed as follows: 0 min, 90% A; 0–40 min, 70% A; 40–60 min, 30% A; 60–64 min, 10% A; 64–70 min, 90% A. The flow rate of 0.5 mL/min and injection volume of 10 µL were set.

Analysis of all triterpenic compounds was carried out on the ACE C18 column (150 mm × 4.6 mm, 3 µm) reversed-phase column (ACT, Aberdeen, UK). For evaluation of triterpenoid acids (maslinic, corosolic, betulinic, oleanolic, and ursolic acids) and neutral triterpenoids with chromophores (betulin and uvaol), isocratic elution consisting of acetonitrile and water (89:11, *v*/*v*) at a flow rate of 0.7 mL/min was used. The column temperature was maintained at 20 °C. Chromatographic separation of neutral triterpenoids, which lack chromophores (betulinic acid methyl ester, α-amyrin, and β-amyrin) and phytosterol (β-sitosterol) was performed at isocratic elution consisting of methanol and acetonitrile (90:10, *v*/*v*), at a flow rate of 1 mL/min when column temperature was set at 35 °C. The injection volume of triterpenoid extracts was 10 µL. 

More data on the methods used were presented in previous papers and in Table S1 [36,49]. 

### 4.5. Antioxidant Activity Assays

The antioxidant activity of *A. millefolium* extracts was analysed using the ABTS radical cation decolourization assay (ABTS) and ferric reducing antioxidant power (FRAP) assays. The ABTS radical cation is described by Re et al. [49]. FRAP was performed using the method described by Benzie and Strain [50] with the modifications as detailed in our previous paper [51,52] using a spectrophotometer (Spectronic CamSpec M550, Garforth, UK). Antiradical and reducing activity results were expressed as micromolar of Trolox equivalents per gram of dry weight of plant material (μmol TE/g, DW).

### 4.6. Statistical Analysis

Results were expressed as the mean ± standard deviation (SD) from three replicates. Significant differences in chemical compounds among populations and plant organs were tested using one-way analysis of variance (ANOVA) at a confidence level of *p* < 0.05, followed by the posthoc Duncan’s Multiple Range tests [53]. Pearson’s correlation analysis was performed to explain the relationship between variables and principal components and the *p*-value obtained by testing the hypothesis on nonlinear regression. The data underwent principal component analysis (PCA), ensuring adequacy through Bartlett’s test [54] of sphericity and the Kaiser–Meyer–Olkin measure of sampling adequacy [55]. Factors with eigenvalues surpassing 1 were taken into consideration. The data were processed using Microsoft Office Excel for 365 Version 2403 (Microsoft, Redmond, WA, USA) and SPSS 29 Version 29.0.1.0 (171) (IBM, Armonk, NY, USA) software.

## 5. Conclusions

Research on *Achillea millefolium* remains relevant due to its potential therapeutic benefits and ecological significance associated with its diverse compounds. This study highlighted the morphological diversity within the species, particularly in inflorescence colour-specific morphotypes. The observed variations in phenolic compounds and triterpenic profiles between morphotypes and plant parts potentially reflect the adaptive nature of *A. millefolium* in response to its local environment and genetic diversity. This study highlighted the chemical differences between *A. millefolium* morphotypes based on inflorescence colour-specific differences. The phytochemical profile of white and pink inflorescence morphotypes of *A. millefolium* was characterised by a complex of thirty-four phenolic and triterpene compounds. Significant differences in morphotype-dependent phytochemical profiles were expressed only for triterpenic compounds. In general, white inflorescences accumulated a higher amount of compounds compared to pink inflorescences. The leaves revealed qualitative differences from other plant parts due to the additional presence of ursolic acid, nicoflorin, isoquercitrin, and cynarin, indicating their higher value. Furthermore, the leaves of both morphotypes have been testified as the main source of antioxidant active compounds.

Notably, our search revealed a lack of data on phenolic and triterpenic profiling in the coloured inflorescence of *A. millefolium* morphotypes, suggesting promising future research to better understand the chemical and morphological diversity of the target species. We assume that the differences in the phytochemical profiles of *A. millefolium* morphotypes were not significantly affected by habitat, as both morphotypes originated from the same population. Differences in specialised metabolites and their chemotypes likely reflect their genetic differences. The morphotype can serve as a marker for the selection of a certain chemotype. Mapping of chemotypic and morphotypic data with pharmacological activity contributes to drug discovery and development and promotes the responsible and evidence-based use of herbal medicines in healthcare.

## Figures and Tables

**Table 1:** Mean quantities (µg/g, DW) of phenolic and triterpenic compounds in the inflorescence, leaf and stem samples of *Achillea milefollium* morphotypes.

Compound	IW ^1^	IP	LW	LP	SW	SP
Oleanolic acid	29.22 ± 14.64 ^2^	80.03 ± 15.35	102.68 ± 44.53	139.25 ± 44.53	0 ± 0	0 ± 0
ursolic	0 ± 0	0 ± 0	16.78 ± 0	0 ± 0	0 ± 0	0 ± 0
Maslinic acid	18.02 ± 5.18	32.96 ± 9.66	32.18 ± 13.71	58.27 ± 13.71	14.31 ± 2.92	16.04 ± 6.26
Corosolic acid	46.1 ± 15.44	78.79 ± 12.14	86.81 ± 3	91.1 ± 3	5.36 ± 0.55	1.39 ± 0.86
Betulinic acid	72.38 ± 18.52	197.27 ± 66.68	440.54 ± 78.94	519.01 ± 51.77	0 ± 0	0 ± 0
Betulin	2327.85 ± 726.91	1114.17 ± 257.8	956.93 ± 110.63	992.32 ± 153.83	1016.3 ± 5.5	888.95 ± 0
Uvaol	24.87 ± 14.45	23.38 ± 6.68	0 ± 0	0 ± 0	0 ± 0	0 ± 0
Betulinic acid methyl ester	226.15 ± 77.43	239.78 ± 75.7	230.81 ± 63.13	225.74 ± 63.13	23.84 ± 6.72	19.12 ± 2.69
beta-Amyrin	571.63 ± 56.64	388.28 ± 97.07	153.61 ± 33.32	115.71 ± 33.32	15.15 ± 3.9	16.31 ± 6.8
beta-Sitosterol	283.77 ± 68.47	211.71 ± 53.62	166.02 ± 50.38	165.21 ± 50.38	118.36 ± 16.33	125.77 ± 31.3
Alfa-amyrin	153.83 ± 37.42	84.95 ± 10.24	297.29 ± 33.4	250.44 ± 33.4	19.41 ± 11.61	9.08 ± 6.67
Nicotiflorin	0 ± 0	0 ± 0	237.18 ± 33.8	138.88 ± 33.8	0 ± 0	0 ± 0
Isovitexin	14.03 ± 4.05	25.18 ± 12.2	27.15 ± 12.46	24.92 ± 8.46	0 ± 0	0 ± 0
Hesperidin	491.5 ± 152.82	670.9 ± 188.85	1104.06 ± 35.51	1380.83 ± 50.58	238.54 ± 113.48	285.37 ± 121.65
Luteolin-7-*O*-glucuronide	2176.98 ± 444.59	1540.17 ± 310.57	5068.57 ± 345.75	3459.83 ± 345.75	611.39 ± 302.49	292.64 ± 199.77
Neochlorogenic acid	402.67 ± 53.65	482.4 ± 61.5	1184.96 ± 99.82	1190.55 ± 99.82	263.4 ± 47.24	268.3 ± 52.92
Chlorogenic acid	7969.08 ± 226.96	7848.88 ± 346.64	28,123.17 ± 1013.01	28,934.28 ± 1864.98	4583.7 ± 579.72	4076.34 ± 333.15
4-*O*-caffeoylquinic acid	1313.7 ± 229.52	212.6 ± 69.91	405.28 ± 393.89	1058.9 ± 393.89	685.87 ± 46.00	96 ± 51.24
3.4-*O*-dicaffeoylquinic acid	2545.78 ± 540.08	2546.82 ± 340.77	3749.7 ± 784.63	3338.91 ± 784.63	651.83 ± 188.26	714.49 ± 184.64
3.5-*O*-dicaffeoylquinic acid	9014.24 ± 732.64	8502.93 ± 762.67	16,064.82 ± 984.88	14,974.35 ± 984.88	1486.04 ± 245.46	1344.03 ± 503.54
1.5-*O*-dicaffeoylquinic acid	2595.3 ± 213.95	2414.05 ± 100.41	4957.9 ± 807.78	5765.53 ± 807.78	1070.17 ± 448.67	932.41 ± 481.21
4.5-*O*-dicaffeoylquinic acid	1005.02 ± 239.56	1310.5 ± 188.35	2075.26 ± 702.21	2402.77 ± 702.21	590.36 ± 230.26	605.63 ± 217.05
Caffeic acid	17.46 ± 7.65	8.48 ± 3.94	64.14 ± 37.48	34.86 ± 17.48	33.93 ± 15.52	12.54 ± 9.12
Quercitrin	174.5 ± 23.63	106.46 ± 19.88	306.19 ± 134.67	229.69 ± 134.67	110.86 ± 56.22	42.19 ± 19.23
Rutin	176.2 ± 84.9	304.7 ± 42.42	2634.46 ± 862.93	2965.2 ± 1397.95	734.99 ± 414.21	1095.84 ± 440.83
Quercetin	27.61 ± 3	27.8 ± 5	18.76 ± 8.01	21.17 ± 8.01	19.83 ± 2.74	19.46 ± 3.05
Isoquercitrin	0 ± 0	0 ± 0	86.01 ± 9.83	137.9 ± 27.87	5.3 ± 0.25	42.76 ± 19.14
Luteolin	110.98 ± 3.13	2685.86 ± 384.43	144.96 ± 27.41	128.29 ± 27.41	137.77 ± 7.54	133.14 ± 5.5
Luteolin-7-*O*-glucoside	2792.46 ± 446.2	2016.26 ± 376.03	1032.26 ± 344.69	749.36 ± 344.69	89.37 ± 34.81	63.85 ± 28.59
Luteolin-7-*O*-rutinoside	756.05 ± 156.91	680.43 ± 99.55	1296.21 ± 655.24	780.66 ± 655.24	232.89 ± 97.13	50.41 ± 34.16
Apigenin	559.24 ± 65.3	413.08 ± 69.63	11.03 ± 2.15	2.49 ± 0.15	0 ± 0	0 ± 0
Apigenin-*O*-7-glucoside	3535.22 ± 462.02	2724.33 ± 338.68	258.94 ± 102.82	184.97 ± 102.82	27.1 ± 11.6	22.21 ± 9.07
Santin	249.57 ± 25.88	220.59 ± 11.77	248.04 ± 84.09	257.24 ± 84.09	207.03 ± 0.93	147.97 ± 93.59
Cynarin	0 ± 0	0 ± 0	74.99 ± 34.59	30.2 ± 34.59	28.49 ± 23.71	2.87 ± 6.75

^1^ IW—White inflorescences; IP—pink inflorescences; LW—white leaves; LP—pink leaves; SW—white stems; SP—pink stems; ^2^ Values are expressed as mean ± SE.

**Table 2:** Description of *Achillea millefolium* collecting locations.

Pop. No	Administrative Location	Latitude (°N)	Longitude (°E)	Elevation (m.a.s.l.)	Habitat
1	Einororys, Alytus distr.	54.44614	24.38971	120	Mesophytic grassland
2	Geruliai, Alytus distr.	54.53120	24.27056	130	Mesophytic grassland
3	Užugriovis, Vilnius distr.	54.82782	25.24644	161	Dry grassland
4	Bernatonys, Vilnius distr.	54.90934	25.32271	160	Mesophytic grassland
5	Vorėnai, Molėtai distr.	55.35779	25.61012	158	Mesophytic grassland
6	Juodžionys, Biržai distr.	56.24353	24.87915	60	Mesophytic grassland
7	Dubingiai, Molėtai distr.	55.05911	25.43509	175	Pine forest, roadside

## Data Availability

All data generated during this study are included in this article.

## Supplementary Materials

The following supporting information can be downloaded at: https://www.mdpi.com/article/10.3390/plants13071043/s1, Table S1: HPLC-PDA methods phenolic and triterpenic compounds identification and quantification parameters.
