# Supplementary material for: Comparative Phytoprofiling of Achillea millefolium Morphotypes: Assessing Antioxidant Activity, Phenolic and Triterpenic Compounds Variation across Different Plant Parts
Source: Plants (Basel). 2024 Apr 8;13(7):1043. doi: 10.3390/plants13071043 (PMC11013869; doi:10.3390/plants13071043)
Supplement: Supplementary file 1 [file plants-13-01043-s001.zip › plants-2916085-supplementary.pdf]

Table S1. HPLC-PDA methods phenolic and triterpenic compounds identification and quantification parameters.

Compound	Calibration curve	Coefficient of determination (r^2)	LOD (µg/mL)	LOQ (µg/mL)
Neochlorogenic acid	y = 44600x - 47800	0.99981	0.37	1.13
Chlorogenic acid	y = 47700x + 2650	0.99992	0.13	0.41
4-*O*-caffeoylquinic acid	y = 60200x - 16200	0.99996	0.08	0.23
3,4-*O*-dicaffeoylquinic acid	y = 56700x - 6790	0.99999	0.03	0.09
3,5-*O*-dicaffeoylquinic acid	y = 79100x - 36900	0.99990	0.09	0.28
1,5-*O*-dicaffeoylquinic acid	y = 70500x - 23900	0.99937	0.65	1.57
4,5-*O*-dicaffeoylquinic acid	y = 40400x - 9600	0.99998	0.05	0.15
Cynarin	y = 58900x + 21100	0.99996	0.35	0.92
Caffeic acid	y = 100000x + 3500	0.99999	0.28	0.86
Quercitrin	y = 30500x + 1380	0.99999	0.08	0.23
Rutin	y = 29000x + 1370	0.99998	0.11	0.32
Hesperidin	y = 408000x +15900	0.99996	0.13	0.35
Quercetin	y = 61900x - 10000	0.99999	0.04	0.13
Isoquercitrin	y = 38400x + 74800	0.99984	0.06	0.19
Nicotiflorin	y = 35800x + 21100	0.99996	0.22	0.71
Luteolin	y = 25200x - 26000	0.99931	0.48	1.44
Luteolin-7-*O*-glucoside	y = 54300x + 1040	0.99998	0.05	0.15
Luteolin-7-*O*-rutinoside	y = 40300x - 3980	0.99999	0.03	0.08
Luteolin-3,7-*O*-diglucoside	y = 31200x + 19100	0.99929	0.31	0.94
Luteolin-7-*O*-glucuronide	y = 49300x + 20600	0.99944	0.25	0.73
Apigenin	y = 90100x + 9770	0.99997	0.03	0.10
Apigenin-7-*O*-glucoside	y = 68600x - 3820	0.99992	0.06	0.17
Santin	y = 64700x - 132000	0.99975	0.47	1.41
Maslinic acid	y = 8960x + 2060	0.99995	0.08	0.24
Corosolic acid	y = 6910x + 1270	0.99991	0.16	0.48
Betulinic acid	y = 8970x + 4310	0.99996	0.11	0.32
Oleanolic acid	y = 12600x + 8710	0.99994	0.21	0.65
Ursolic acid	y = 9040x + 30900	0.99998	0.26	0.82
Betulin	y = 10600x + 4350	0.99999	0.29	0.89
Uvaol	y = 9310x + 4390	0.99993	0.30	0.99
Betulinic acid methyl ester	y = 5940x - 3590	0.99941	0.19	0.61
β-Amyrin	y = 7870x + 4310	0.99999	0.14	0.43
β-Sitosterol	y = 3980x + 3610	0.99992	0.37	1.13
α-Amyrin	y = 6470x + 9440	0.99999	0.24	0.73
